# Supplementary material for: Neuromodulation of the cerebellum rescues movement in a mouse model of ataxia
Source: Nat Commun. 2021 Feb 26;12:1295. doi: 10.1038/s41467-021-21417-8 (PMC7910465; doi:10.1038/s41467-021-21417-8)
Supplement: Supplementary file 1 — Supplementary Information [file 41467_2021_21417_MOESM1_ESM.pdf]

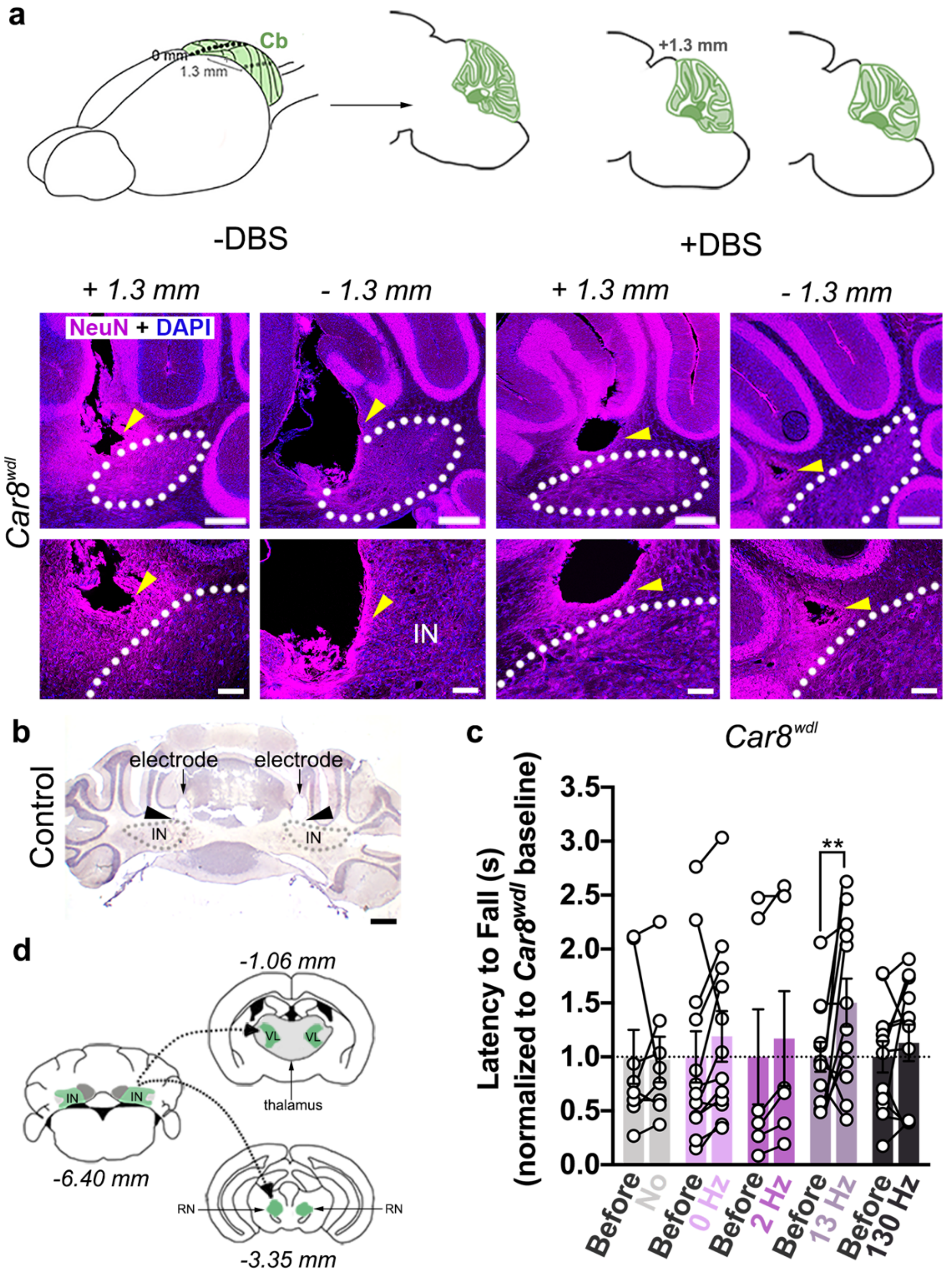
a
Cb
0 mm
1.3 mm
+1.3 mm
-DBS
+DBS
+ 1.3 mm
- 1.3 mm
+ 1.3 mm
- 1.3 mm
NeuN + DAPI
Car8wdl
IN
b
Control
electrode
electrode
IN
IN
c
Car8wdl
Latency to Fall (s)
(normalized to Car8wdl baseline)
**
Before
No
Before
0 Hz
Before
2 Hz
Before
13 Hz
Before
130 Hz
d
-1.06 mm
VL
VL
thalamus
IN
IN
-6.40 mm
RN
RN
-3.35 mm

Supplementary Figure 1. Interposed nucleus targeting is consistent between stimulated and non-stimulated *Car8*^{wdl} mice and its stimulation only elicits improvements at 13 Hz. (a) Schematics detailing the stimulation coordinates on a whole-mount cerebellum (Cb) and the subsequent sagittal sections by which electrode tracks could be located. Below the schematics are examples of *Car8*^{wdl} brain tissue that contain electrode tracks (yellow arrowheads). The interposed nucleus (IN; outlined with white dotted lines) of stimulated and non-stimulated *Car8*^{wdl} mice was bilaterally and accurately targeted. n=6 biologically independent animals (-DBS: n=3; +DBS: n=3) over 6 independent experiments. (b) A coronal view of our bilateral targeting into the cerebellar IN. Black arrowheads point to the end of the electrode tracks. Gray dotted lines outline the IN of the cerebellum. (c) Expanded view of the quantification performed for **Fig. 1**. Only stimulating the IN at 13 Hz results in significant behavioral improvements for *Car8*^{wdl} mice (p=0.0095). A black dotted line demarcates the mutant baseline (normalized to y=1.00). The latency to fall values for each *Car8*^{wdl} mouse were plotted, before and during ±DBS. n=50 biologically independent animals (No Surgery: n=8; 0 Hz: n=12; 2 Hz: n=6; 13 Hz: n=12; 130 Hz: n=12) over 50 independent experiments. (d) Schematics showing two of the primary projections of the interposed cerebellar nuclei (IN): the ventrolateral thalamic nuclei (VL) and the red nucleus (RN). The scale bars in top panels of (a) represent 50 µm. The scale bars in the bottom panels of (a) represent 25 µm. The scale bar in (b) represents 500 µm. ** p<0.01; Two-Way ANOVA, repeated measures; Sidak's multiple comparisons test; Mean ± SEM.

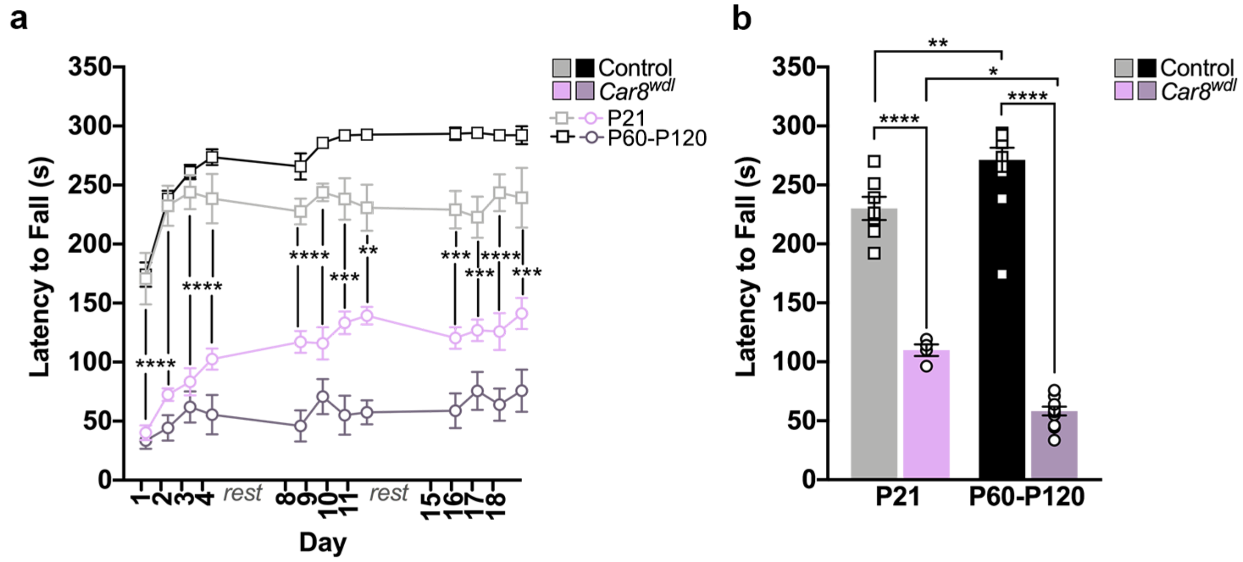

Supplementary Figure 2. *Ca8*^{wdl} mice are noticeably ataxic, starting at P21. (a-b) *Car8*^{wdl} mice perform significantly worse on the rotarod than that of control mice (P21: p<0.0001; P60-P120: p<0.0001). n=29 biologically independent animals (P21: Control, n=7; *Car8*^{wdl}, n=4; P60-P120: Control, n=10; *Car8*^{wdl}, n=8) over 29 independent experiments in (a). n=26 biologically independent animals (P21: Control, n=7; *Car8*^{wdl}, n=4; P60-P120: Control, n=7; *Car8*^{wdl}, n=8) over 26 independent experiments in (b). Expanded dataset detailed in **Supplementary Data 1**. * p<0.05; ** p<0.01; *** p<0.001; **** p<0.0001; Two-Way ANOVA, repeated measures with mixed-effects (a); Unpaired two-tailed Student's t-test and One-Way ANOVA (b); Tukey's multiple comparisons test (a); Dunnett's multiple comparisons test (b); Mean ± SEM.

a

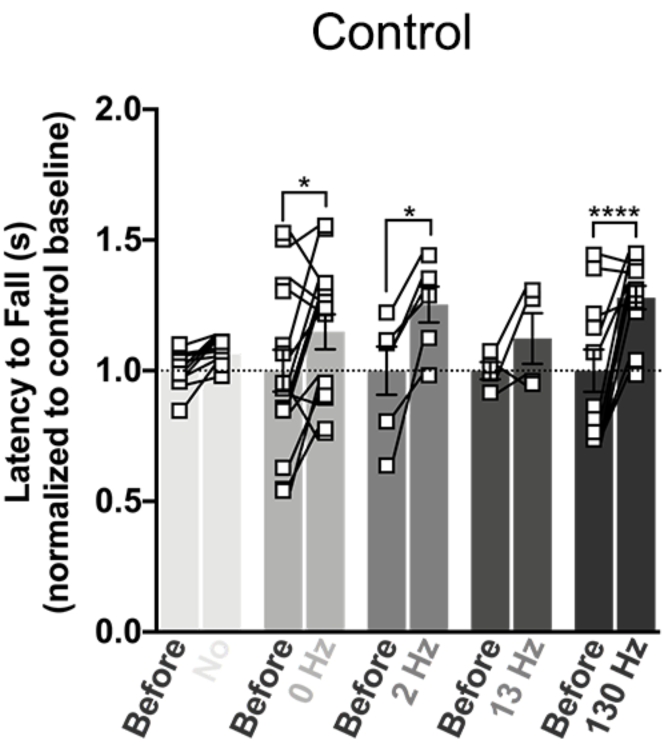

b

± DBS

Days 12-16

P60-P120 Control

Hz	% Improvement
No	6.58 ± 2.01%
0	20.43 ± 7.00%
2	28.94 ± 9.57%
13	12.12 ± 8.08%
130	33.79 ± 8.34%

Supplementary Figure 3. Control mice modestly improve with cerebellar DBS. (a-b) Control mice minimally improve from their baselines with stimulation, with the effect only being significant when behavioral deficits (e.g., due to surgical lesions) exist prior to stimulation (No Surgery: $p=0.9463$; 0 Hz: $p=0.0321$; 2 Hz: $p=0.0166$; 13 Hz: $p=0.8414$; 130 Hz: $p<0.0001$). A black dotted line demarcates the control baseline (normalized to y=1.00). The latency to fall values for each control mouse were plotted, before and during ±DBS. n=46 biologically independent animals (No Surgery: n=10; 0 Hz: n=15; 2 Hz: n=6; 13 Hz: n=4; 130 Hz: n=11) over 46 independent experiments. * $p<0.05$; **** $p<0.0001$; Two-Way ANOVA, repeated measures; Sidak's multiple comparisons test; Mean ± SEM.

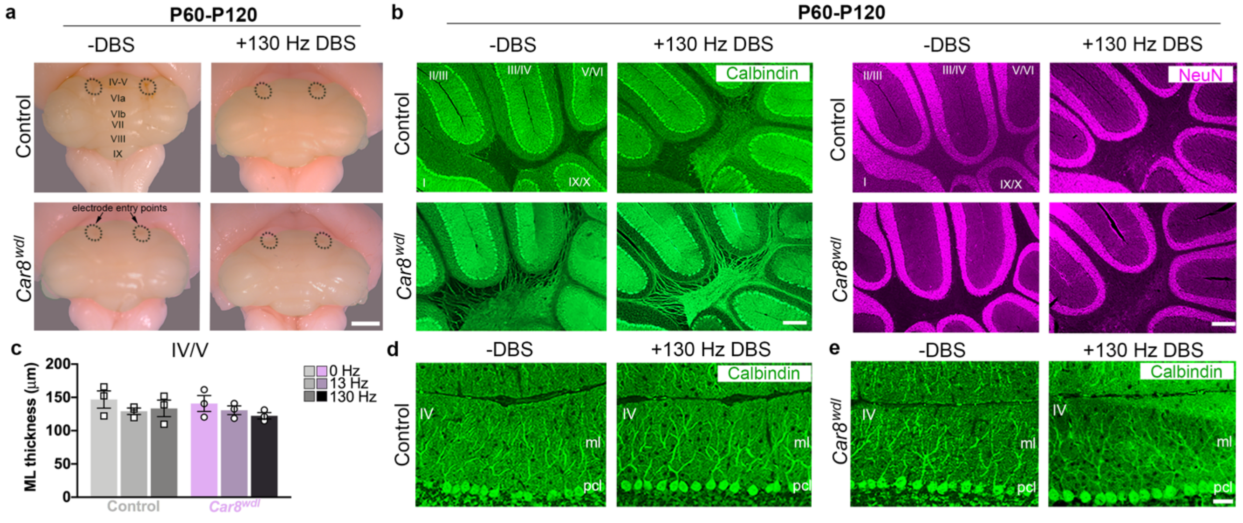

Supplementary Figure 4. DBS and sham surgeries have similar effects on cerebellar anatomy. (a) Gross cerebellar morphology is undisturbed in the presence and absence of DBS. Black dotted lines outline the electrode locations. (b) Cerebellar circuit anatomy is preserved in stimulated and non-stimulated mice. Calbindin (green) labels Purkinje cells whereas NeuN (magenta) labels granule cells in the cerebellum. (c) The molecular layer (ML) thickness of lobules IV/V, a key locomotion-relevant region in the cerebellum, is comparable between genotypes (Control, 0/13 Hz: p=0.7790; Control, 0/130 Hz: p=0.9146; Control 13/130 Hz: p=0.9994; *Car8*^{wdl}, 0/13 Hz: p=0.9712; *Car8*^{wdl}, 0/130 Hz: p=0.7496; *Car8*^{wdl}, 13/130 Hz: p=0.9982) and across DBS paradigms (0 Hz: p=0.9973; 13 Hz: p>0.9999; 130 Hz: p=0.9585). (d-e) Representative images from lobule IV of stimulated and non-stimulated control and mutant mice showing that Purkinje cell cytoarchitecture is preserved. ml = molecular layer; pcl = Purkinje cell layer. The scale bar represents 1000 µm in (a), 200 µm in (b), and 50 µm in (d-e). n=18 biologically independent animals (-DBS: Control, n=3; *Car8*^{wdl}, n=3; 13 Hz: Control, n=3; *Car8*^{wdl}, n=3; 130 Hz: Control, n=3; *Car8*^{wdl}, n=3) over 18 independent experiments. Two-Way ANOVA; Tukey's multiple comparisons test; Mean ± SEM.

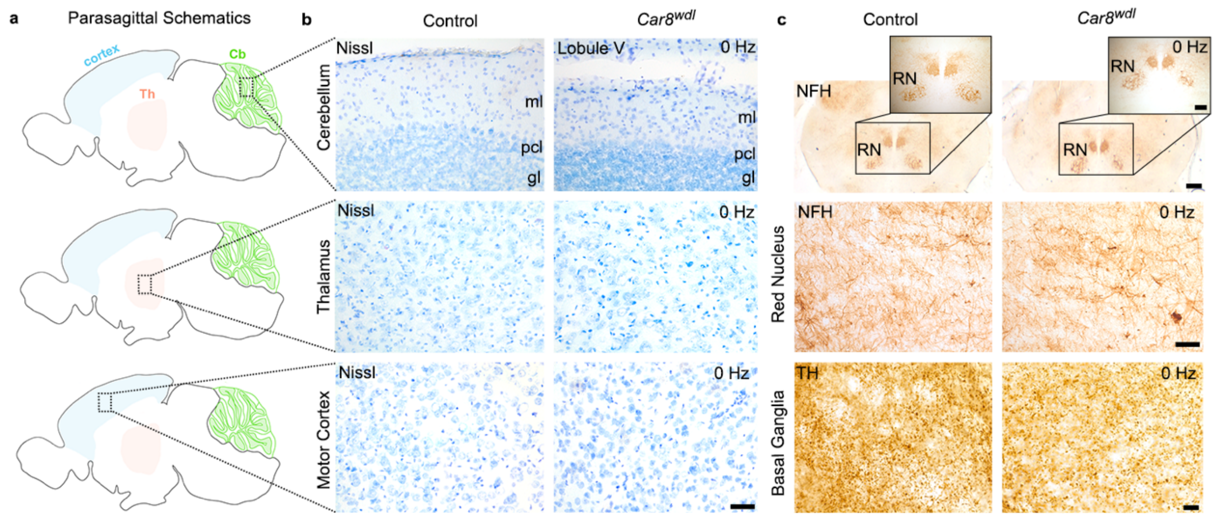

Supplementary Figure 5. *Car8*^{wdl} motor circuity is intact after surgery. (a) Parasagittal schematics of the tissue sections collected. Key nodes of the motor circuit are colored in green (cerebellum, Cb), salmon (thalamus, Th), and blue (cortex). (b) Nissl nuclear staining reveals similar cellular counts in the cerebellum, thalamus, and cortex between control and mutant mice. ml = molecular layer; pcl = Purkinje cell layer; gl = granule layer. Scale bar represents 50 µm. (c) Neurofilament Heavy Chain (NFH) staining reveals that the red nucleus (RN) is morphologically similar in control and mutant mice. Scale bars represent 500 µm (zoomed out) and 200 µm (zoomed in) on the coronal sections. Sagittal sectioning of the brain tissue also reveals comparative NFH staining in the RN. Staining for tyrosine hydroxylase (TH) shows that the mutant mice have dopaminergic profiles in the basal ganglia, which is essential for executing movement. Scale bar for the sagittal sections stained with NFH and TH represents 50 µm and 10 µm, respectively. n=6 biologically independent animals (Control: n=3; *Car8*^{wdl}: n=3) over 6 independent experiments.

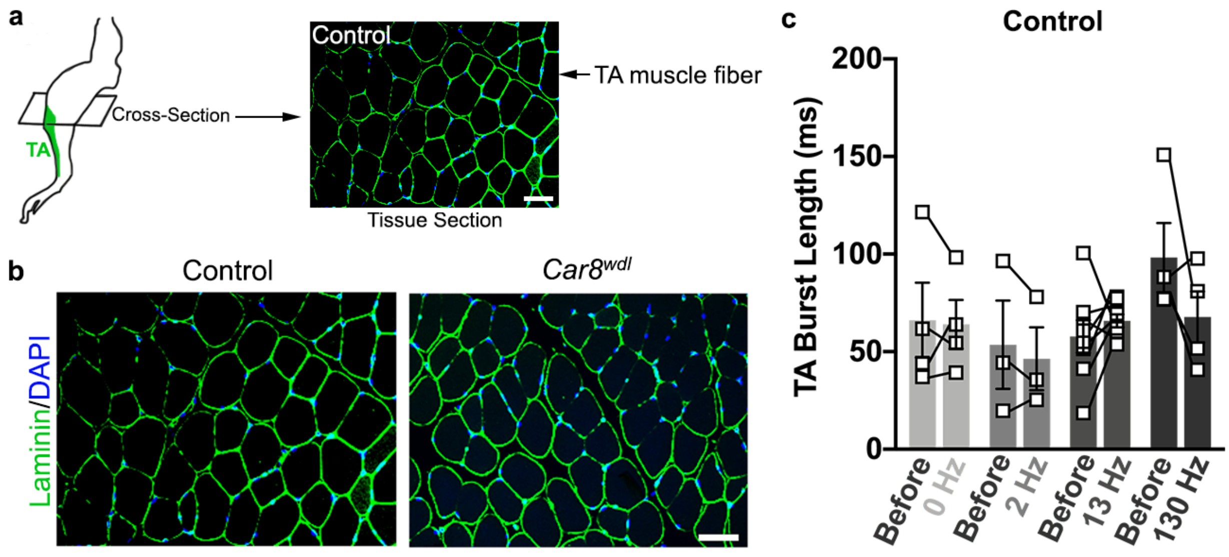

Supplementary Figure 6. Muscle anatomy and function are preserved after surgery and stimulation. (a) The tibialis anterior (TA, green) muscle of the left hindlimb from control and mutant mice was cut sagittally in order to visualize the individual muscle fibers. (b) There is no atrophy or degeneration in the *Car8*^{wdl} TA muscle. The scale bar represents 50 µm. Laminin (green) outlines individual muscle fibers of the TA. DAPI (blue) labels the nuclei. n=6 biologically independent animals (Control: n=3; *Car8*^{wdl}: n=3) over 6 independent experiments. (c) DBS does not significantly alter the TA burst length of control mice (0 Hz: p=0.9995; 2 Hz: p=0.9718; 13 Hz: p=0.8007; 130 Hz: p=0.0646). n=19 biologically independent animals (0 Hz: n=4; 2 Hz: n=3; 13 Hz: n=8; 130 Hz: n=4) over 19 independent experiments. Two-Way ANOVA, repeated measures; Sidak's multiple comparisons test; Mean ± SEM.

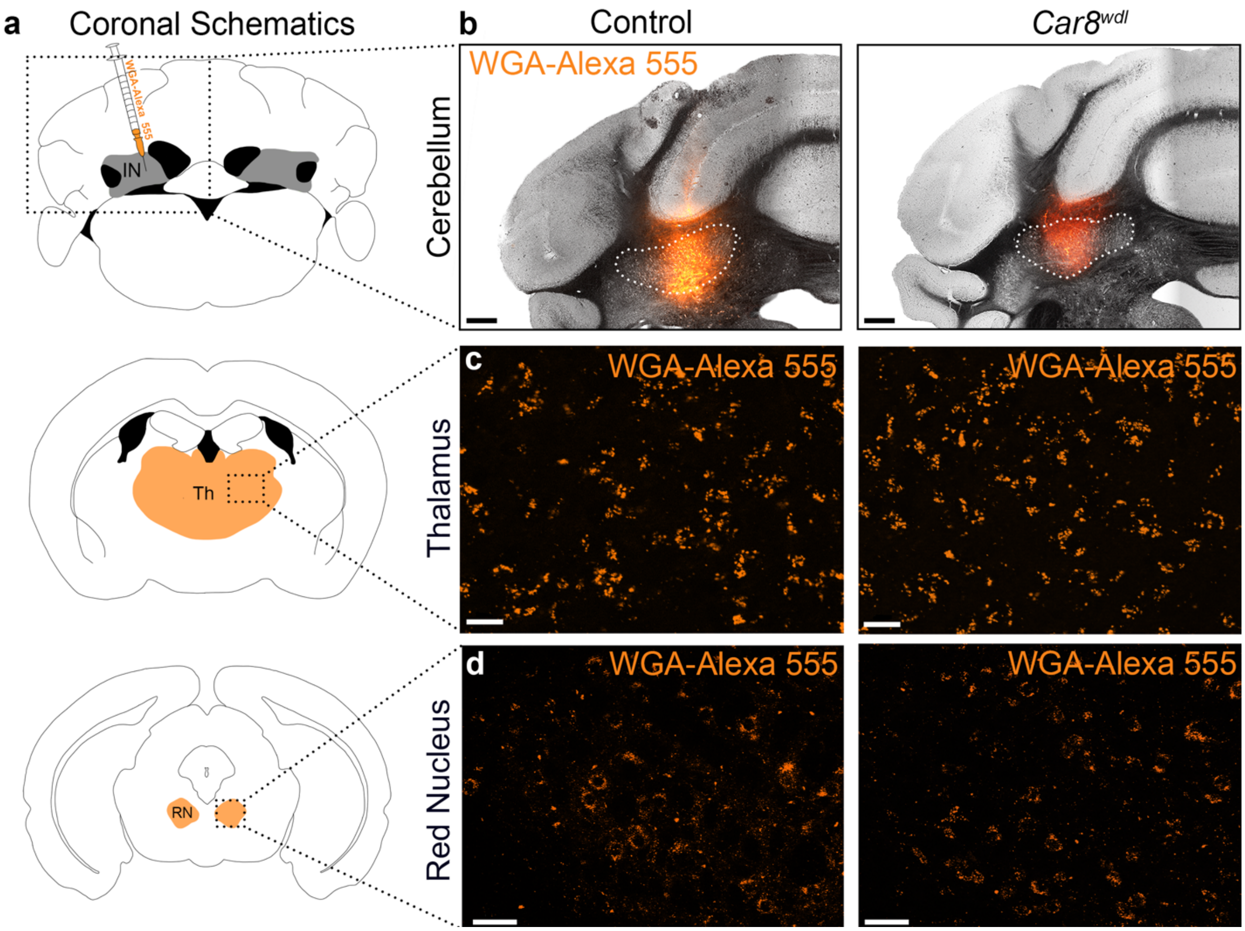

Supplementary Figure 7. Cerebellar output projections are comparable between control and *Car8*^{wdl} mutant mice. (a) Coronal schematics of the tissue sections collected. Downstream targets of the interposed cerebellar nucleus (IN) are colored orange. Th = thalamus; RN = red nucleus. (b) Representative images showing WGA-Alexa 555 targeting to the interposed nucleus (IN) of control and *Car8*^{wdl} mutant mice. (c-d) Cerebellar projections to the thalamus (c) and red nucleus (d) are preserved as anterogradely labeled terminals are evident in *Car8*^{wdl} mice. The scale bars represent 500 µm (b), 20 µm (c), and 50 µm (d). n=6 biologically independent animals (Control: n=3; *Car8*^{wdl}: n=3) over 6 independent experiments.

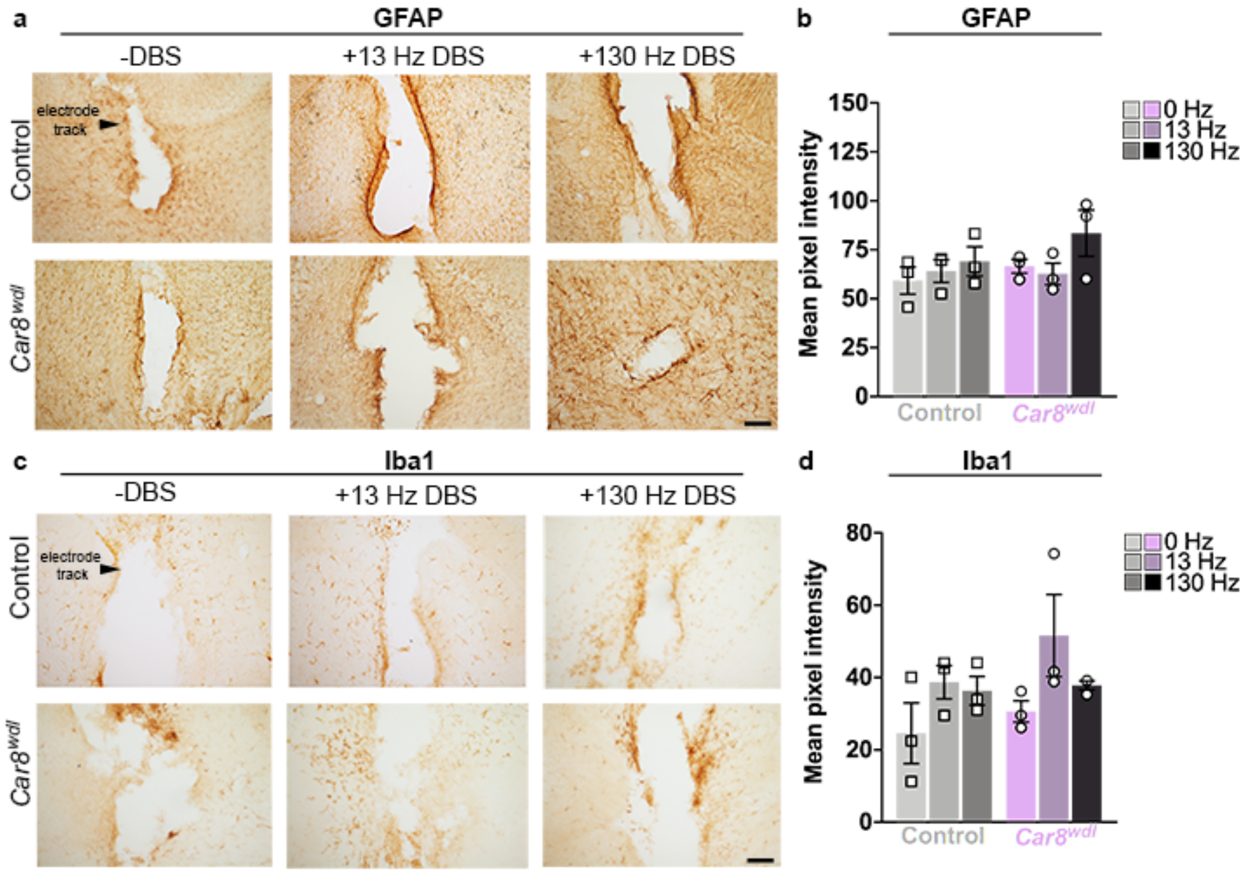

Supplementary Figure 8. Neuroinflammatory responses are consistent across DBS paradigms. (a-b) Glial scarring (GFAP) is comparable between genotypes (Control, 0/13 Hz: p>0.9999; Control, 0/130 Hz: p=0.9988; Control 13/130 Hz: p>0.9999; *Car8*^{wdl}, 0/13 Hz: p>0.9999; *Car8*^{wdl}, 0/130 Hz: p=0.8704; *Car8*^{wdl}, 13/130 Hz: p=0.6455) and stimulation paradigms (0 Hz: p>0.9999; 13 Hz: p>0.9999; 130 Hz: p=0.9556). (c-d) Inflammation (Iba1) is comparable between genotypes (Control, 0/13 Hz: p=0.9069; Control, 0/130 Hz: p=0.9760; Control 13/130 Hz: p>0.9999; *Car8*^{wdl}, 0/13 Hz: p=0.4507; *Car8*^{wdl}, 0/130 Hz: p=0.9998; *Car8*^{wdl}, 13/130 Hz: p=0.9189) and stimulation paradigms (0 Hz: p>0.9999; 13 Hz: p=0.9486; 130 Hz: p>0.9999). The scale bars represent 50 µm. n=18 biologically independent animals (Control, -DBS: n=3; Control, 13 Hz: n=3; Control, 130 Hz: n=3; *Car8*^{wdl}, -DBS: n=3; *Car8*^{wdl}, 13 Hz: n=3; *Car8*^{wdl}, 130 Hz: n=3) over 18 independent experiments. Two-Way ANOVA; Sidak's multiple comparisons test; Mean ± SEM.

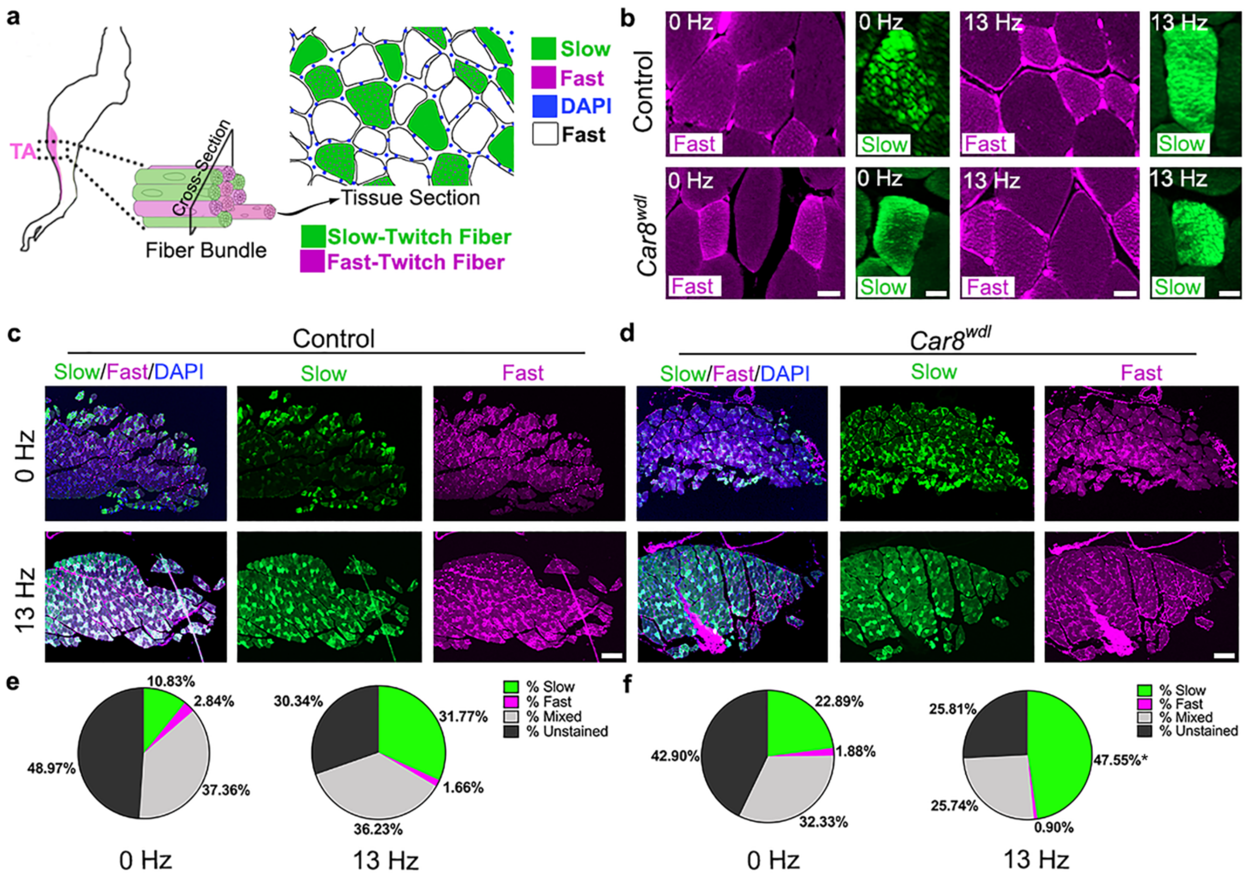
a
TA
Cross-Section
Fiber Bundle
Tissue Section
Slow
Fast
DAPI
Fast
Slow-Twitch Fiber
Fast-Twitch Fiber
b
Control
Car8wdl
0 Hz
13 Hz
Fast
Slow
c
Control
d
Car8wdl
Slow/Fast/DAPI
Slow
Fast
0 Hz
13 Hz
e
10.83%
2.84%
37.36%
48.97%
0 Hz
30.34%
31.77%
1.66%
36.23%
13 Hz
% Slow
% Fast
% Mixed
% Unstained
f
22.89%
1.88%
32.33%
42.90%
0 Hz
25.81%
47.55%*
0.90%
25.74%
13 Hz

Supplementary Figure 9. Cerebellar DBS increases the expression of slow-twitch muscle proteins. (a) Schematic of the intact mouse TA muscle and its cross-section. Magenta represents fast-twitch myofibrils while green represents slow-twitch myofibrils. In the cross-section, staining for fast- and slow-twitch muscle proteins reveals fibers that are either slow (green), fast (magenta), putatively fast (unstained), or mixed (green and magenta). (b-d) 13 Hz cerebellar DBS increases slow-twitch protein expression in both the control and mutant TA muscle, without overtly affecting fast-twitch protein expression. (e-f) The percent of slow-twitch fibers increases after 13 Hz stimulation in control and *Car8*^{wdl} mice, although the changes are only statistically significant in the mutants (Control, 13 Hz: p=0.0787; *Car8*^{wdl}, 13 Hz: p=0.0312). The scale bars represent 50 µm. n=12 biologically independent animals (Control, 0 Hz: n=3; Control, 13 Hz: n=3; *Car8*^{wdl}, 0 Hz: n=3; *Car8*^{wdl}, 13 Hz: n=3) over 12 independent experiments. * $p<0.05$; Three-Way ANOVA (e-f); Sidak's multiple comparisons test.

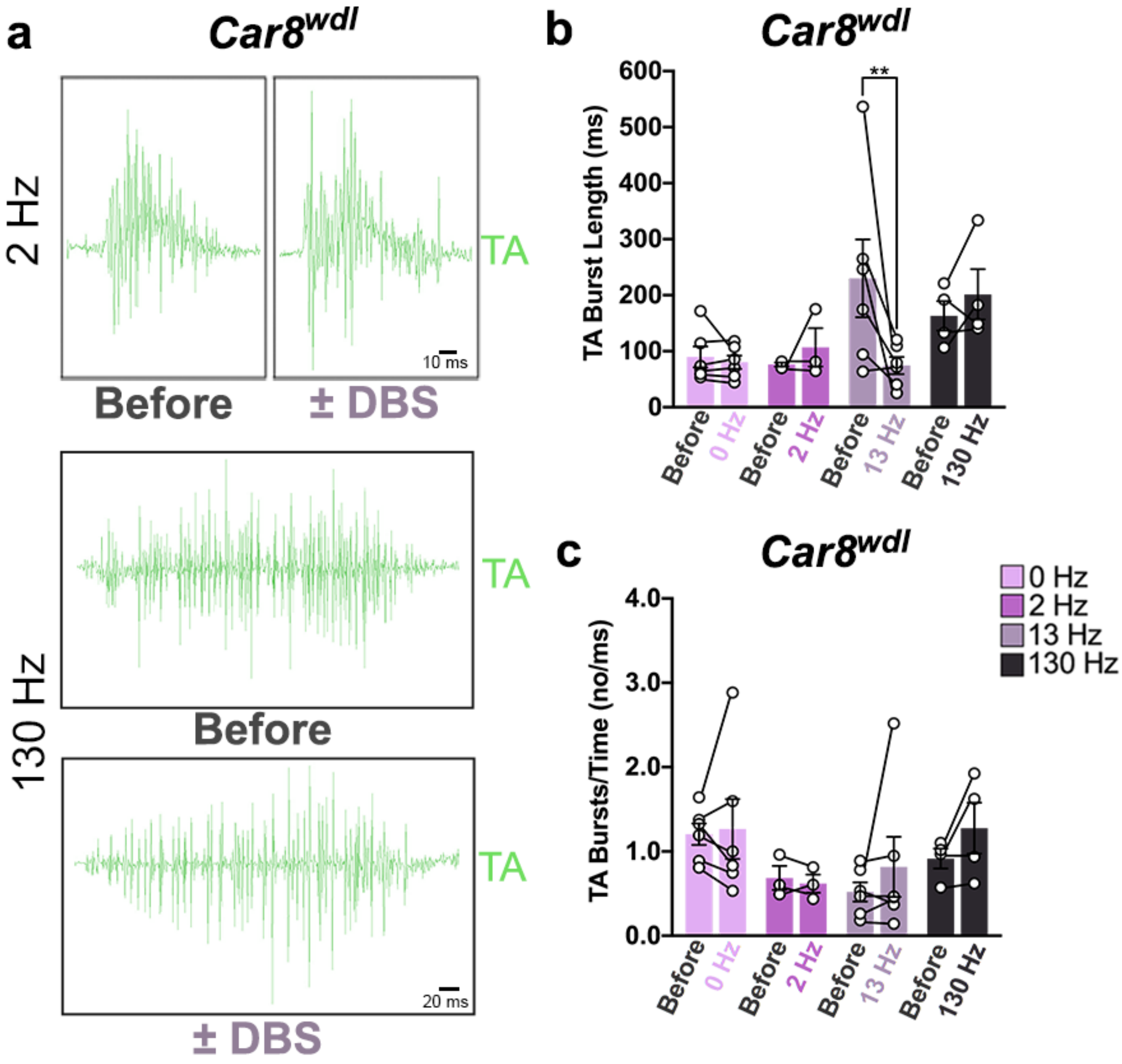
a
Car8wdl
2 Hz
TA
10 ms
Before
± DBS
130 Hz
TA
Before
TA
20 ms
± DBS
b
Car8wdl
TA Burst Length (ms)
600
500
400
300
200
100
0
**
Before
0 Hz
Before
2 Hz
Before
13 Hz
Before
130 Hz
c
Car8wdl
0 Hz
2 Hz
13 Hz
130 Hz
TA Bursts/Time (no/ms)
4.0
3.0
2.0
1.0
0.0
Before
0 Hz
Before
2 Hz
Before
13 Hz
Before
130 Hz

Supplementary Figure 10. Improved TA responses depends on the DBS frequency. (a) DBS delivered at 2 or 130 Hz does not improve TA muscle firing in *Car8*^{wdl} mice. (b) Quantification summarizing the effects of cerebellar stimulation on TA muscle firing in *Car8*^{wdl} mice (0 Hz: p=0.9988; 2 Hz: p=0.9731; 13 Hz: p=0.0058; 130 Hz: p=0.9052). n=19 biologically independent animals (0 Hz: n=6; 2 Hz: n=3; 13 Hz: n=6; 130 Hz: n=4) over 19 independent experiments. (c) No stimulation paradigm changes the frequency of TA bursting in *Car8*^{wdl} mice (0 Hz: p=0.9984; 2 Hz: p=0.9994; 13 Hz: p=0.6495; 130 Hz: p=0.6569). n=19 biologically independent animals (0 Hz: n=6; 2 Hz: n=3; 13 Hz: n=6; 130 Hz: n=4) over 19 independent experiments. ** p<0.01; Two-Way ANOVA, repeated measures; Sidak's multiple comparisons test; Mean ± SEM.

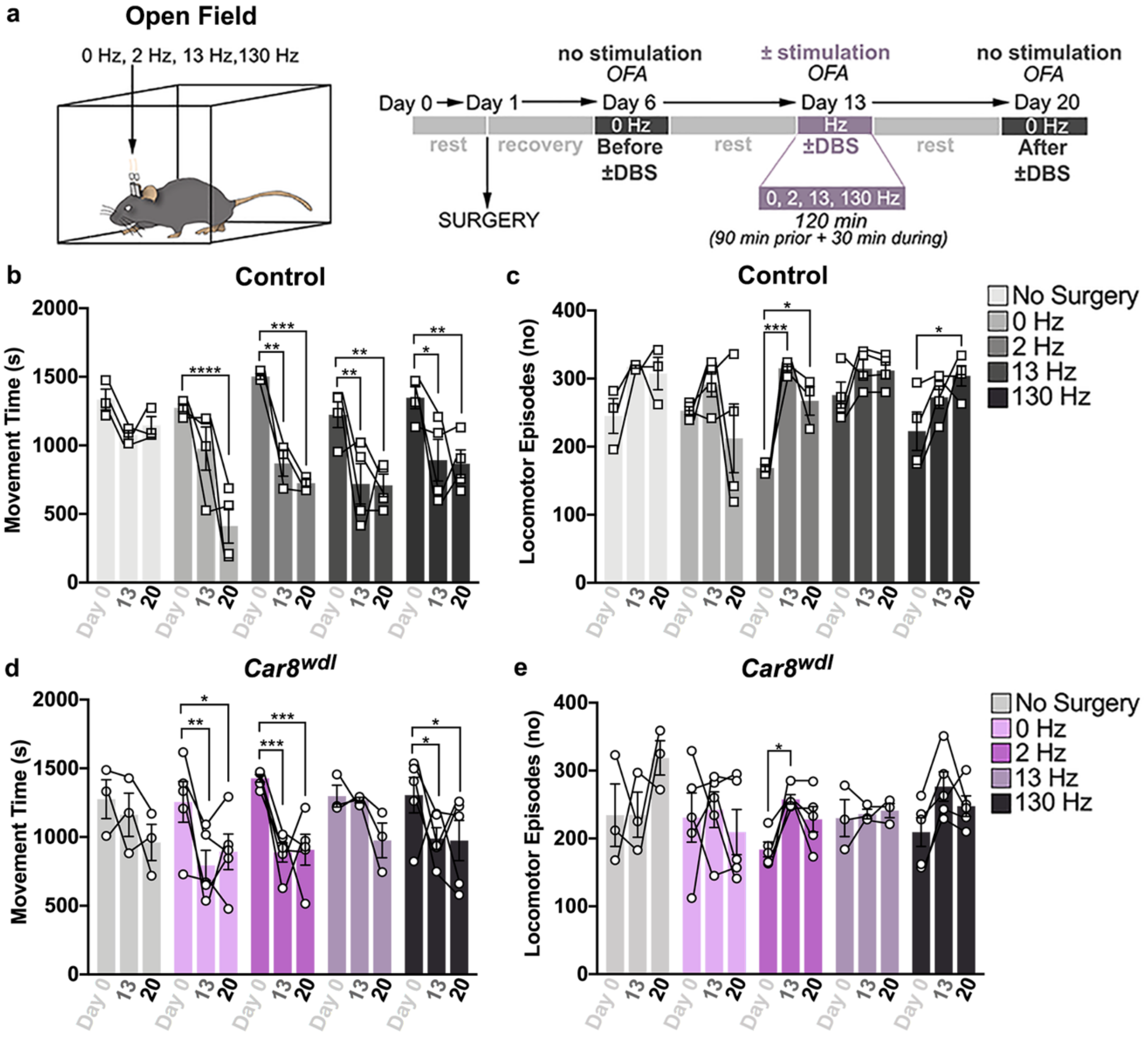

f — Control

Hz	Time Day 13	Time Day 20	Episodes Day 13	Episodes Day 20
No	n.s.	n.s.	n.s.	n.s.
0	n.s.	****	n.s.	n.s.
2	**	***	***	*
13	**	**	n.s.	n.s.
130	*	**	n.s.	*

g — *Car8^wdl*

Hz	Time Day 13	Time Day 20	Episodes Day 13	Episodes Day 20
No	n.s.	n.s.	n.s.	n.s.
0	**	*	n.s.	n.s.
2	***	***	*	n.s.
13	n.s.	n.s.	n.s.	n.s.
130	*	*	n.s.	n.s.

Supplementary Figure 11. Quantification and summary of data acquired from combining DBS with the open field assay (OFA). (a) Schematic and experimental timeline of the OFA behavioral test used in combination with DBS to measure movement. (b) Control mice move for less time when stimulated at all frequencies (0 Hz: Day 20, p<0.0001; 2 Hz: Day 13, p=0.0028, Day 20, p=0.0004; 13 Hz: Day 13, p=0.0058, Day 20, p=0.0049; 130 Hz: Day 13, p=0.0125, Day 20, p=0.0082). n=18 biologically independent animals (No Surgery: n=3; 0 Hz: n=4; 2 Hz: n=3; 13 Hz: n=4; 130 Hz: n=4) over 18 independent experiments. (c) 2 and 130 Hz cerebellar stimulation impacts the number of times control mice move in 30 minutes (2 Hz: Day 13, p=0.0006, Day 20, p=0.0175; 130 Hz: Day 20, p=0.0240). no = number. n=18 biologically independent animals (No Surgery: n=3; 0 Hz: n=4; 2 Hz: n=3; 13 Hz: n=4; 130 Hz: n=4) over 18 independent experiments. (d) Most stimulation frequencies impede $Car8^{wdl}$ movement (0 Hz: Day 13, p=0.0017, Day 20, p=0.0134; 2 Hz: Day 13, p=0.0003, Day 20, p=0.0005; 130 Hz: Day 13, p=0.0324, Day 20, p=0.0249), except for 13 Hz (Day 13, p=0.9771, Day 20, p=0.1008), over the 20-day experimental period. n=21 biologically independent animals (No Surgery: n=3; 0 Hz: n=5; 2 Hz: n=5; 13 Hz: n=3; 130 Hz: n=5) over 21 independent experiments. (e) 2 Hz DBS impacts the number of times mutant mice move in 30 minutes (p=0.0396). no = number. n=21 biologically independent animals (No Surgery: n=3; 0 Hz: n=5; 2 Hz: n=5; 13 Hz: n=3; 130 Hz: n=5) over 21 independent experiments. (f-g) Summary tables detailing how each stimulation paradigm affects movement time and the number of locomotor episodes in control (f) and mutant (g) mice. Day 0 = Before Surgery. Day 13 = ±DBS. Day 20 = After ±DBS. * p<0.05; ** p<0.01; *** p<0.001; **** p<0.0001; n.s. = not significant; Two-Way ANOVA, repeated measures; Dunnett's multiple comparisons test; Mean ± SEM.

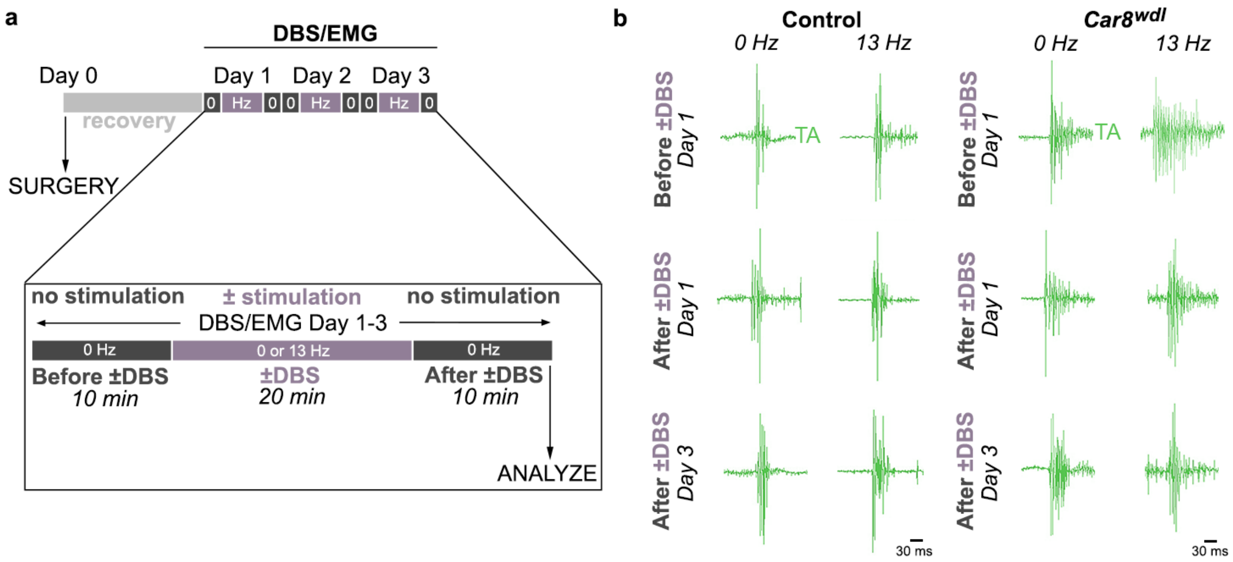

Supplementary Figure 12. 13 Hz DBS further suppresses TA pathology in *Car8*^{wdl} mice after 3 days. (a) Experimental timeline detailing the stimulation paradigm followed. TA burst activity was analyzed 10 minutes after DBS was stopped. (b) Representative EMG traces showing TA bursts. TA burst length is significantly reduced after 3 days of 13 Hz DBS in *Car8*^{wdl} mice. TA burst length is unaltered in 0 Hz- and 13 Hz-stimulated control mice and in 0 Hz-stimulated *Car8*^{wdl} mice. n=24 biologically independent animals (Control, 0 Hz: n=4; Control, 13 Hz: n=8; *Car8*^{wdl}, 0 Hz: n=6; *Car8*^{wdl}, 13 Hz: n=6) over 24 independent experiments on Day 1. n=15 biologically independent animals (Control, 0 Hz: n=3; Control, 13 Hz: n=3; *Car8*^{wdl}, 0 Hz: n=4; *Car8*^{wdl}, 13 Hz: n=5) over 15 independent experiments on Day 3.

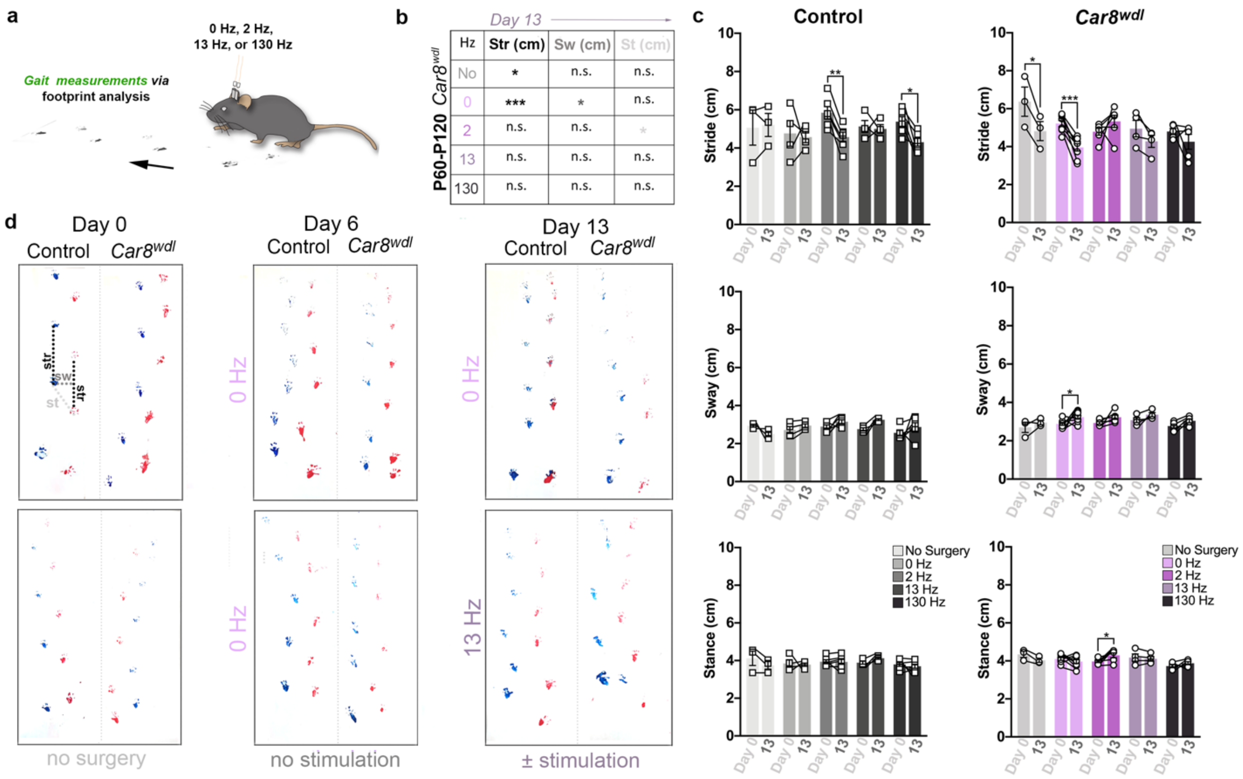
a
0 Hz, 2 Hz, 13 Hz, or 130 Hz
Gait measurements via footprint analysis
b
Day 13
P60-P120 Car8wdl
Hz | Str (cm) | Sw (cm) | St (cm)
No | * | n.s. | n.s.
0 | *** | * | n.s.
2 | n.s. | n.s. | [illegible]
13 | n.s. | n.s. | n.s.
130 | n.s. | n.s. | n.s.
c
Control
Car8wdl
Stride (cm)
Sway (cm)
Stance (cm)
Day 0
13
No Surgery
0 Hz
2 Hz
13 Hz
130 Hz
d
Day 0
Day 6
Day 13
Control
Car8wdl
str
sw
st
0 Hz
13 Hz
no surgery
no stimulation
± stimulation

Supplementary Figure. 13. Cerebellar DBS preserves the overall gait of *Car8*^{wdl} mice. (a) Schematic of combining footprint analysis with DBS to study gait. (b) Summary of the *Car8*^{wdl} gait changes that accompany time, surgery, and stimulation (2, 13, 130 Hz). The gait of P60-P120 *Car8*^{wdl} mice is naturally variable over time (Str: p=0.0106) and worsens with 0 Hz (Str: p=0.0007; Sw: p=0.0384) or 2 Hz of stimulation (St: p=0.0365). Str = stride; Sw = sway; St = stance. (c) Stride is impacted by stimulation in control mice (2 Hz: p=0.0032; 130 Hz: p=0.0134), but not in *Car8*^{wdl} mice (2 Hz: p=0.3985; 13 Hz: p=0.3959; 130 Hz: p=0.5061). Sway and stance are largely unchanged in both genotypes (Sw: Control, No, p=0.2847; 0 Hz, p=0.7626; 2 Hz, p=0.4799; 13 Hz, p=0.0981; 130 Hz, p=0.2784; *Car8*^{wdl}, No, p=0.6945; 2 Hz, p=0.1906; 13 Hz, p=0.4297; 130 Hz, p=0.3515; St: Control, No, p=0.2062; 0 Hz, p=0.9965; 2 Hz, p>0.9999; 13 Hz, p=0.5091; 130 Hz, p=0.9407; *Car8*^{wdl}, No, p=0.2482; 0 Hz, p=0.5743; 13 Hz, p=0.9926; 130 Hz, p=0.7201). (d) Examples of footprint traces prior to surgery (Day 0), after surgery (Day 6), and with or without DBS (Day 13). *Car8*^{wdl} mice that are stimulated at 13 Hz maintain their gait. n=49 biologically independent animals (Control, No Surgery: n=3; Control, 0 Hz: n=4; Control, 2 Hz: n=6; Control, 13 Hz: n=4; Control, 130 Hz: n=6; *Car8*^{wdl}, No Surgery: n=3; *Car8*^{wdl}, 0 Hz: n=8; *Car8*^{wdl}, 2 Hz: n=6; *Car8*^{wdl}, 13 Hz: n=4; *Car8*^{wdl}, 130 Hz: n=5) over 49 independent experiments. * p<0.05; ** p<0.01; *** p<0.001; n.s. = not significant; Two-Way ANOVA, repeated measures; Sidak's multiple comparisons test; Mean ± SEM.

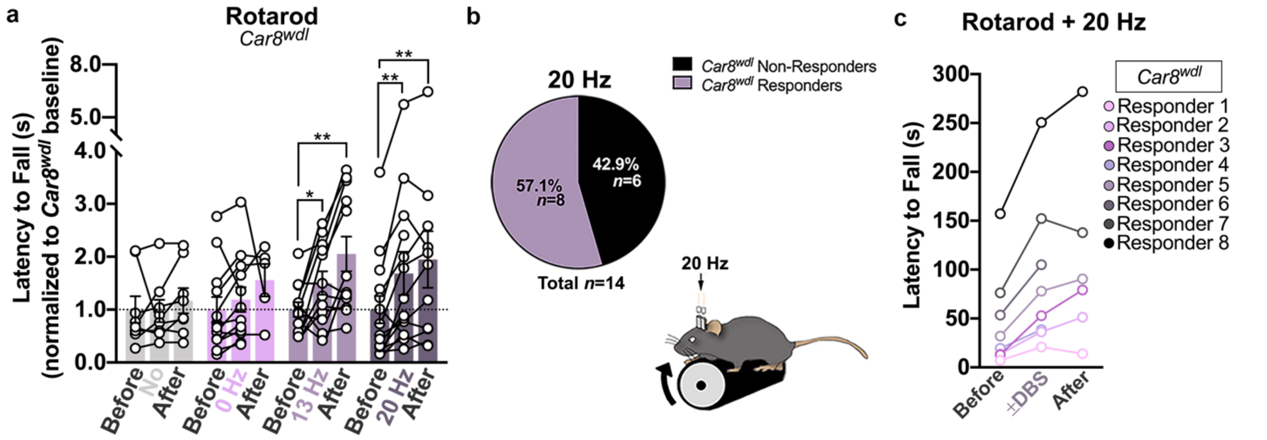

Supplementary Figure 14. Beneficial DBS outcomes occur at beta-frequencies. (a) *Car8^{wdl}* mice significantly improve on the rotarod after 13 (p=0.0055) and 20 Hz DBS (p=0.0062). n=46 biologically independent animals (No Surgery: n=8; 0 Hz: n=12; 13 Hz: n=12; 20 Hz: n=14) over 46 independent "Before ±DBS" and "±DBS" experiments. n=36 biologically independent animals (No Surgery: n=8; 0 Hz: n=5; 13 Hz: n=12; 20 Hz: n=11) over 36 independent "After ±DBS" experiments. (b) 57.1% of 20 Hz-stimulated *Car8^{wdl}* mice (n=8/14) improve on the rotarod. (c) Improvements depend on the motor function of *Car8^{wdl}* mice prior to 20 Hz stimulation. Each line represents a *Car8^{wdl}* "responder" (n=8). * p<0.05; ** p<0.001; Two-Way ANOVA, repeated measures with mixed-effects (a); Dunnett's multiple comparisons test (a); Mean ± SEM (a).

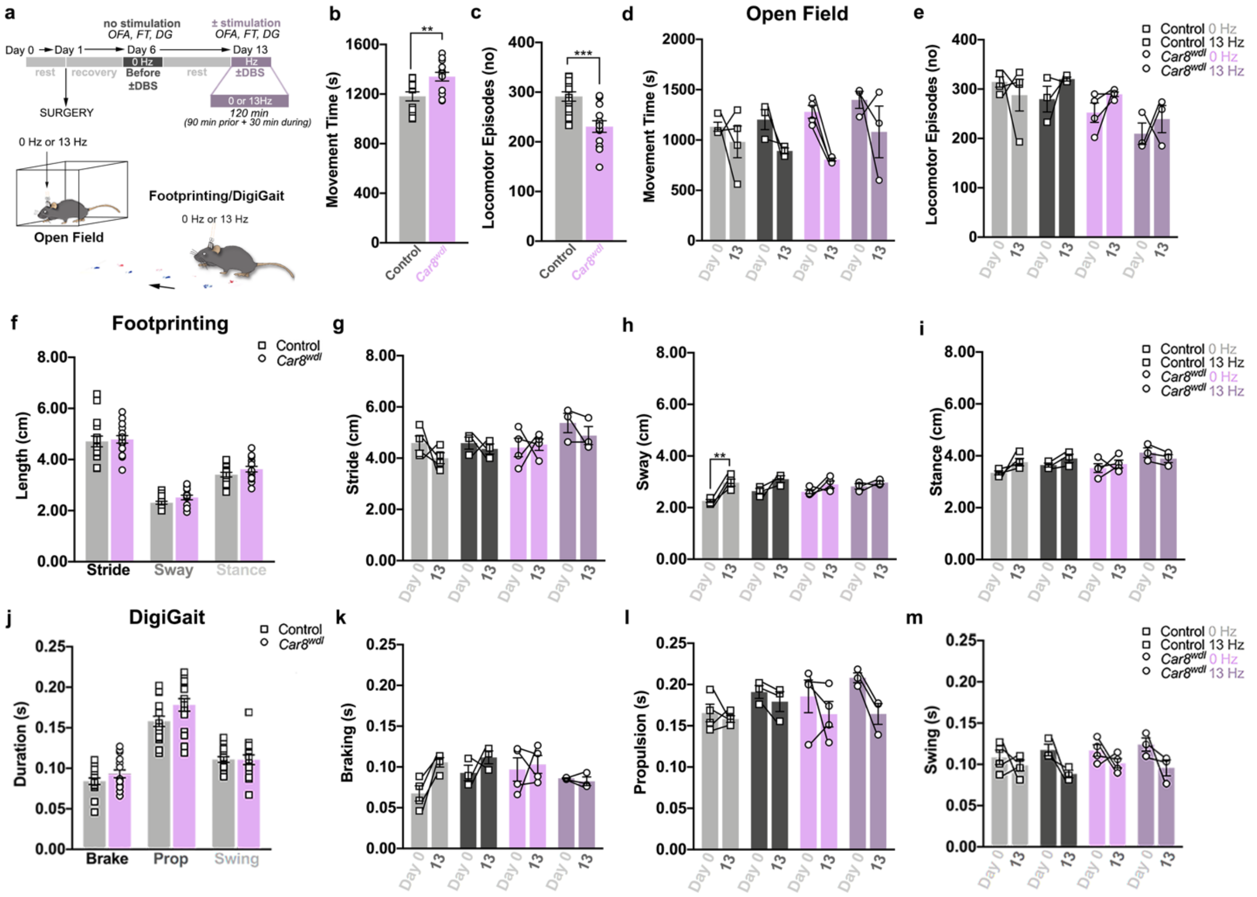
a
no stimulation
OFA, FT, DG
± stimulation
OFA, FT, DG
Day 0
Day 1
Day 6
Day 13
rest
recovery
0 Hz
Before
±DBS
rest
Hz
±DBS
0 or 13Hz
120 min
(90 min prior + 30 min during)
SURGERY
0 Hz or 13 Hz
Open Field
Footprinting/DigiGait
0 Hz or 13 Hz
b
Movement Time (s)
Control
Car8wdl
c
Locomotor Episodes (no)
Control
Car8wdl
d
Open Field
Movement Time (s)
e
Locomotor Episodes (no)
Control 0 Hz
Control 13 Hz
Car8wdl 0 Hz
Car8wdl 13 Hz
f
Footprinting
Length (cm)
Control
Car8wdl
Stride
Sway
Stance
g
Stride (cm)
h
Sway (cm)
i
Stance (cm)
Control 0 Hz
Control 13 Hz
Car8wdl 0 Hz
Car8wdl 13 Hz
j
DigiGait
Duration (s)
Control
Car8wdl
Brake
Prop
Swing
k
Braking (s)
l
Propulsion (s)
m
Swing (s)
Control 0 Hz
Control 13 Hz
Car8wdl 0 Hz
Car8wdl 13 Hz
Day 0
13

Supplementary Figure 15. DBS does not worsen movement or gait in P30 *Car8*^{wdl} mice. (a) The experimental timeline followed, and schematics of the behavioral assays used with P30 control and *Car8*^{wdl} mice. OFA = open field assay; FT = footprinting; DG = DigiGait. (b-c) P30 *Car8*^{wdl} mice move more (p=0.0045) and stop less (p=0.0005) than P30 control mice. n=26 biologically independent animals (Control: n=13; *Car8*^{wdl}: n=13) over 26 independent experiments. (d-e) Surgical intervention reduces P30 *Car8*^{wdl} hypermobility (0 Hz: n=4, p=0.0699; 13 Hz: n=3, p=0.4325). no = number. n=14 biologically independent animals (Control, 0 Hz: n=4; Control, 13 Hz: n=3; *Car8*^{wdl}, 0 Hz: n=4; *Car8*^{wdl}, 13 Hz: n=3) over 14 independent experiments. (f) P30 *Car8*^{wdl} mice do not exhibit gait changes (Stride: p=0.7638; Stance: p=0.1430; Sway: p=0.0626). n=32 biologically independent animals (Control: n=16; *Car8*^{wdl}: n=16) over 32 independent experiments. (g-i) Surgical intervention does not worsen *Car8*^{wdl} gait at P30 (Stride: 0 Hz, n=4, p=0.9985; 13 Hz, n=3, p=0.8343; Stance: 0 Hz, p=0.8836; 13 Hz: p=0.7682; Sway: 0 Hz, p=0.3366; 13 Hz, p=0.8870). n=14 biologically independent animals (Control, 0 Hz: n=4; Control, 13 Hz: n=3; *Car8*^{wdl}, 0 Hz: n=4; *Car8*^{wdl}, 13 Hz: n=3) over 14 independent experiments. (j) P30 *Car8*^{wdl} mice do not exhibit kinematic deficits (Braking: p=0.1539; Propulsion: p=0.0525; Swing: p=0.9696). n=32 biologically independent animals (Control: n=16; *Car8*^{wdl}: n=16) over 32 independent experiments. (k-m) Surgical intervention does not worsen *Car8*^{wdl} gait kinematics at P30 (Braking: 0 Hz, p=0.9859; 13 Hz, p=0.9989; Propulsion: 0 Hz, p=0.5212; 13 Hz, p=0.0974; Swing: 0 Hz, p=0.6638; 13 Hz, p=0.2692). n=14 biologically independent animals (Control, 0 Hz: n=4; Control, 13 Hz: n=3; *Car8*^{wdl}, 0 Hz: n=4; *Car8*^{wdl}, 13 Hz: n=3) over 14 independent experiments. ** p<0.01; ***p<0.001; Unpaired, two-tailed Student's t-test (b-c, f, j); Three-Way ANOVA, repeated measures (d-e, g-i, k-m); Sidak's multiple comparisons test (d-e, g-i, k-m); Mean ± SEM.

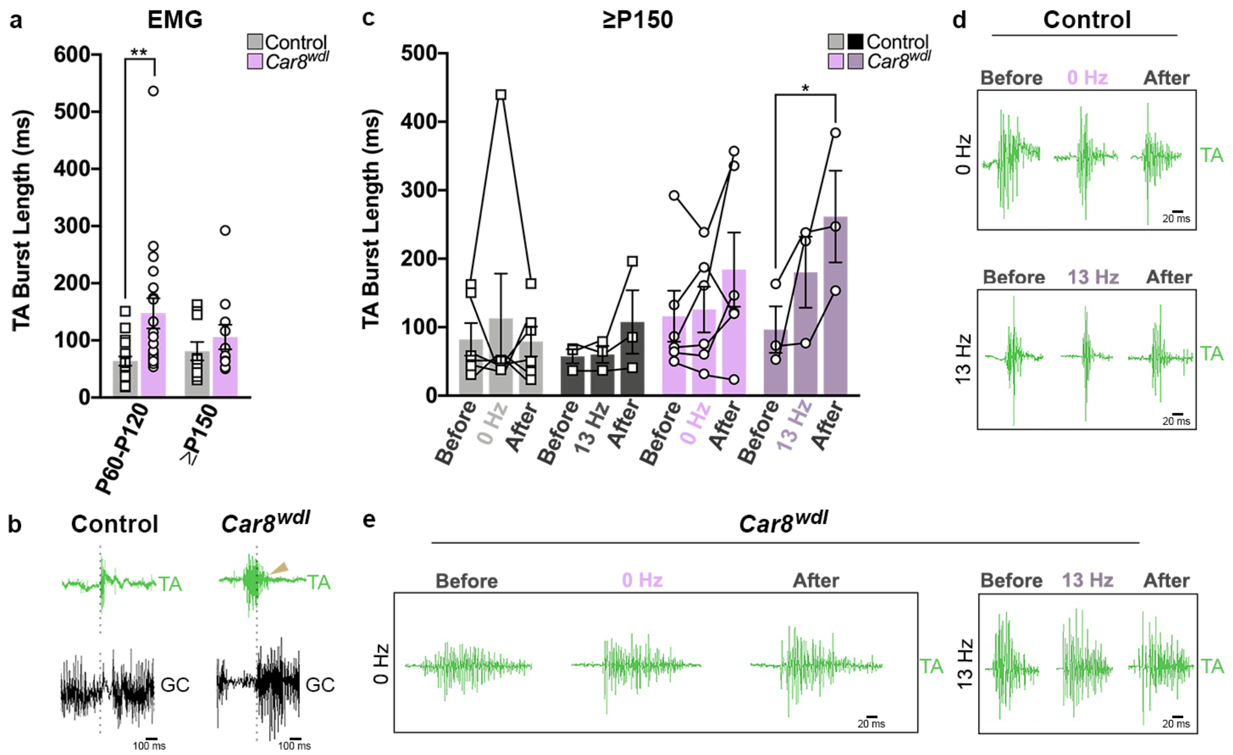
a
EMG
Control
Car8wdl
TA Burst Length (ms)
600
500
400
300
200
100
0
**
P60-P120
≥P150
c
≥P150
Control
Car8wdl
TA Burst Length (ms)
500
400
300
200
100
0
*
Before
0 Hz
After
Before
13 Hz
After
Before
0 Hz
After
Before
13 Hz
After
d
Control
Before
0 Hz
After
0 Hz
TA
20 ms
Before
13 Hz
After
13 Hz
TA
20 ms
b
Control
Car8wdl
TA
TA
GC
GC
100 ms
100 ms
e
Car8wdl
Before
0 Hz
After
0 Hz
TA
20 ms
Before
13 Hz
After
13 Hz
TA
20 ms

Supplementary Figure 16. Age does not significantly alter *Car8*^{wdl} TA firing but affects muscle responsiveness to DBS. (a) *Car8*^{wdl} TA burst length is unaltered with age (p=0.2927), but *Car8*^{wdl} TA muscles function similarly to that of controls at ≥P150 (p=0.3738). n=59 biologically independent animals (P60-P120 Control: n=19; ≥P150 Control: n=10; P60-P120 *Car8*^{wdl}: n=19; ≥P150 *Car8*^{wdl}: n=11) over 59 independent experiments. (b) Example EMG traces from stimulated and non-stimulated control and *Car8*^{wdl} mice. *Car8*^{wdl} TA and GC activities still overlap during locomotion. TA = tibialis anterior; GC = gastrocnemius. (c) DBS significantly prolongs TA activity in ≥P150 *Car8*^{wdl} mice (13 Hz: p=0.4208, After 13 Hz: p=0.0465). n=18 biologically independent animals (≥P150 Control, 0 Hz: n=6; ≥P150 Control, 13 Hz: n=3; ≥P150 *Car8*^{wdl}, 0 Hz: n=6; ≥P150 *Car8*^{wdl}, 13 Hz: n=3) over 18 independent animals. (d-e) Representative EMG traces showing TA bursts from non-stimulated and 13 Hz-stimulated mice. TA burst length is unaltered in 0 Hz (0 Hz: p=0.8835; After 0 Hz: p>0.9999) and 13 Hz-stimulated (13 Hz: p>0.9999; After 13 Hz: p=0.8661) ≥P150 control mice (d) and in 0 Hz-stimulated (0 Hz: p=0.9699, After 0 Hz: p=0.6296) ≥P150 *Car8*^{wdl} mice (e). TA burst length significantly worsens with 13 Hz DBS in aged *Car8*^{wdl} mice (e). * p<0.05; ** p<0.01; Unpaired, two-tailed Student's t-test (a); Three-Way ANOVA, repeated measures (c); Sidak's multiple comparisons test (c); Mean ± SEM (a,c).

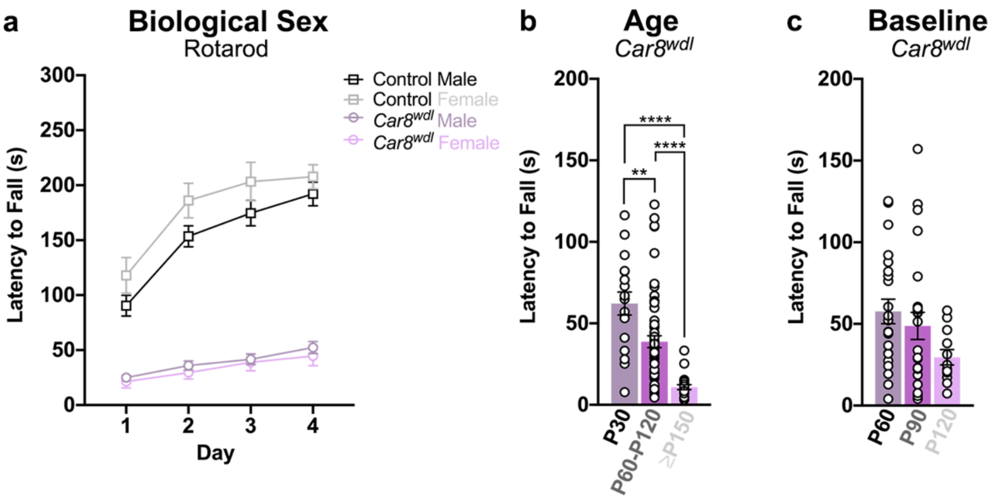

Supplementary Figure 17. Cohorts were made based on age and not biological sex. (a) There are no differences in how male and female mice perform on the rotarod after surgery (Day 4: Control, p=0.8801; *Car8^{wdl}*, p=0.8057). Therefore, biological sex did not decide the treatment groups. n=114 biologically independent animals (Control, male: n=35; Control, female: n=22; *Car8^{wdl}*, male: n=43; *Car8^{wdl}*, female: n=14) over 114 independent experiments. (b) P30 *Car8^{wdl}* mice perform significantly better than P60-P120 (p=0.0010) and ≥P150 *Car8^{wdl}* mice (p<0.0001) and P60-P120 *Car8^{wdl}* mice perform significantly better than ≥P150 *Car8^{wdl}* mice (p<0.0001) after surgery and during the rotarod training period (Days 1-4). Therefore, mutant mice were separated into P30, P60-P120, and ≥P150 experimental treatment groups. n=104 biologically independent animals (P30: n=17; P60-P120: n=65; ≥P150: n=22) over 104 independent experiments. (c) P60, P90, and P120 *Car8^{wdl}* mice establish similar baselines (Days 3-4) on the rotarod (P60 versus P90: p=0.6825; P60 versus P120: p=0.1138; P90 versus P120: p=0.3580). n=64 biologically independent animals (P60: n=26; P60-P120: n=27; ≥P150: n=11) over 64 independent experiments. ** p<0.01; ****p<0.0001. Two-Way ANOVA, repeated measures (a); One-Way ANOVA; Tukey's multiple comparisons test; Mean ± SEM.
