# Supplementary material for: Neuromodulation of the cerebellum rescues movement in a mouse model of ataxia
Source: Nat Commun. 2021 Feb 26;12:1295. doi: 10.1038/s41467-021-21417-8 (PMC7910465; doi:10.1038/s41467-021-21417-8)
Supplement: Supplementary file 3 — Description of Additional Supplementary Files [file 41467_2021_21417_MOESM3_ESM.pdf]

Description of Additional Supplementary Files

Supplementary Data 1: A summary of the data and statistical tests used throughout the study.

Supplementary Data 2: List of the primer sequences used for genotyping *Car8*, $L7^{Cre}$;$Vgat^{flox/flox}$, and control (C57BLKSJ, $Vgat^{flox/flox}$) mice.

Supplementary Movie 1: $Car8^{wdl}$ mice improve on the accelerating rotarod with 13 Hz cerebellar stimulation.

Supplementary Movie 2: $Car8^{wdl}$ mice perform poorly on the accelerating rotarod when the cerebellum is not stimulated (0 Hz).

Supplementary Movie 3: Control mice perform successfully on the accelerating rotarod, even after electrode implantation (0 Hz).

Supplementary Movie 4: 13 Hz DBS induces long-lasting improvements in $Car8^{wdl}$ motor behaviour.
