# Supplementary material for: Neuromodulation of the cerebellum rescues movement in a mouse model of ataxia
Source: Nat Commun. 2021 Feb 26;12:1295. doi: 10.1038/s41467-021-21417-8 (PMC7910465; doi:10.1038/s41467-021-21417-8)
Supplement: Supplementary file 10 — Reporting Summary [file 41467_2021_21417_MOESM10_ESM.pdf]

nature research

Corresponding author(s): Dr. Roy V. Sillitoe

Last updated by author(s): January 17, 2021

Reporting Summary

Nature Research wishes to improve the reproducibility of the work that we publish. This form provides structure for consistency and transparency in reporting. For further information on Nature Research policies, see Authors & Referees and the Editorial Policy Checklist .

Statistics

For all statistical analyses, confirm that the following items are present in the figure legend, table legend, main text, or Methods section.

n/a	Confirmed	
☐	☒	The exact sample size (n) for each experimental group/condition, given as a discrete number and unit of measurement
☐	☒	A statement on whether measurements were taken from distinct samples or whether the same sample was measured repeatedly
☐	☒	The statistical test(s) used AND whether they are one- or two-sided<br>*Only common tests should be described solely by name; describe more complex techniques in the Methods section.*
☐	☒	A description of all covariates tested
☐	☒	A description of any assumptions or corrections, such as tests of normality and adjustment for multiple comparisons
☐	☒	A full description of the statistical parameters including central tendency (e.g. means) or other basic estimates (e.g. regression coefficient) AND variation (e.g. standard deviation) or associated estimates of uncertainty (e.g. confidence intervals)
☐	☒	For null hypothesis testing, the test statistic (e.g. F, t, r) with confidence intervals, effect sizes, degrees of freedom and P value noted<br>*Give P values as exact values whenever suitable.*
☒	☐	For Bayesian analysis, information on the choice of priors and Markov chain Monte Carlo settings
☒	☐	For hierarchical and complex designs, identification of the appropriate level for tests and full reporting of outcomes
☒	☐	Estimates of effect sizes (e.g. Cohen's d, Pearson's r), indicating how they were calculated

Our web collection on statistics for biologists contains articles on many of the points above.

Software and code

Policy information about availability of computer code

Data collection	Spike2 Software (Version 7.09), Zeiss AxioVision Software (Release 4.8), Zeiss Zen Pro Software with Apotome and Z-Stack features (Version 2.0.0, 2012 edition), DigiGait Imager (Version 16), Accuscan Fusion Software (Version 3.7), MC Stimulus II (Version 3.4.4)
Data analysis	MATLAB (Version R2018b), MS Excel (Version 16.29.1, 19091700), Prism8 Software (Version 8.4.3, 471), ImageJ (Version 1.0), ZapStudio (Version 23.10.2017), Free MP4 Converter (Version 6.2.23), Free Video to JPG Converter (Version 5.0.101), Spike2 Software (Version 7.09)

For manuscripts utilizing custom algorithms or software that are central to the research but not yet described in published literature, software must be made available to editors/reviewers. We strongly encourage code deposition in a community repository (e.g. GitHub). See the Nature Research guidelines for submitting code & software for further information.

Data

Policy information about availability of data

All manuscripts must include a data availability statement. This statement should provide the following information, where applicable:

- Accession codes, unique identifiers, or web links for publicly available datasets
- A list of figures that have associated raw data
- A description of any restrictions on data availability

Data and Materials Availability: All data and materials used are available in the main text or as supplementary materials. The source data underlying Fig. 1e-f, 2f, 3c,e, 4c-g, 5a-d, 6e-f, h-i, and Supplementary Figs. 1c, 2a-b, 3a-b, 4c, 6c, 8b,d, 9e-f, 10b-c, 11b-e, 13c, 14a-c, 15b-m, 16b-c, 17a-c are provided as a Source Data file. The authors will also provide data and materials upon request.

Field-specific reporting

Please select the one below that is the best fit for your research. If you are not sure, read the appropriate sections before making your selection.

Life sciences study design

All studies must disclose on these points even when the disclosure is negative.

Sample size	Sample size was not determined using a priori power analysis but was based on the statistical criteria for significance in observations, as described by Dell et al. (2012). Dell et al. (2012) suggests estimating sample size from experience when experiments are based on the success and/or failure of a method (in our case, DBS) because inherent and unpredictable variability in the design prevents accurately calculating n via a priori power analysis.
Data exclusions	All data points were included in this study unless the electrodes were mistargeted, as determined through post-hoc tissue analyses, or if the subject(s) could not complete the full behavioral paradigms (e.g. Fig. S2b).
Replication	At least 3 mice comprised each experimental group (n≥3). We define each mouse as comprising an independent experiment. Samples included male and female mice of the ages P30, P60-P120, and ≥P150. For rotarod and footprinting, multiple trials were performed by each individual animal, then averaged. All of our results were substantiated with appropriate control experiments, including with relevant litter-mate control mice. All attempts at replication were successful, unless the DBS electrodes were mistargeted.
Randomization	Control and mutant *Car8^{wdl}* mice were randomly assigned to various experimental groups (no surgery, 0, 2, 13, 20, 130 Hz). Each group contained males and females as well as mice of each age ±10 days (P30, P60-P120, ≥P150). Data was processed in groups based on motor performance.
Blinding	Investigators were not blinded during data collection since the subjects had to be stimulated (or not stimulated) at specific frequencies. However, data analyses were performed blinded to the intervention.

Reporting for specific materials, systems and methods

We require information from authors about some types of materials, experimental systems and methods used in many studies. Here, indicate whether each material, system or method listed is relevant to your study. If you are not sure if a list item applies to your research, read the appropriate section before selecting a response.

Materials & experimental systems

n/a	Involved in the study	
☐	☒	Antibodies
☒	☐	Eukaryotic cell lines
☒	☐	Palaeontology
☐	☒	Animals and other organisms
☒	☐	Human research participants
☒	☐	Clinical data

Methods

n/a	Involved in the study	
☒	☐	ChIP-seq
☒	☐	Flow cytometry
☒	☐	MRI-based neuroimaging

Antibodies

Antibodies used

The following antibodies were used in this study:

Calbindin D-28K monoclonal mouse antibody (Swant, Catalog #300)
NeuN anti-rabbit antibody (EMD Millipore, Catalog ABN78)
Iba1 anti-rabbit antibody (Fujifilm Wako, Catalog 019-19741)
GFAP anti-rabbit antibody (DAKO Catalog #Z0334)
Neurofilament heavy chain (NFH) anti-mouse antibody (Covance, Catalog #PCK-592P)
Tyrosine hydroxylase (TH) anti-rabbit antibody (Millipore Sigma, Catalog AB152)
Anti-slow skeletal myosin heavy chain anti-mouse antibody (Abcam, Catalog ab11083)
Anti-fast myosin skeletal heavy chain anti-rabbit antibody (Abcam, Catalog ab91506)
Laminin anti-rabbit antibody (Abcam, Catalog ab11575)
Alexa 488-immunoglobin (Invitrogen Molecular Probes Inc., Eugene, OR, USA, Catalog #A-21202)
Alexa 555- immunoglobin (Invitrogen Molecular Probes Inc., Eugene, OR, USA, Catalog #A-31572)
Alexa 647- immunoglobin (Invitrogen Molecular Probes Inc., Eugene, OR, USA, Catalog #A-31573)
Goat anti-mouse HRP-conjugated antibody (Dako, Catalog P044701-2)
Goat anit-rabbit HRP-conjugated antibody (Dako, Catalog P044901-2)
DAPI, Vectashield Anti-Fade Mounting Medium with DAPI (Vector Laboratories, Catalog #H-1200)

Validation

We have validated most of the primary antibodies used in this study in our own prior publications by performing stains on positive and/or negative control tissue and brain structures. Please see White et al. (2014) for Calbindin D-28K, Brown et al. (2019) for NeuN, White and Sillitoe (2013) for NFH, and White et al. (2014, 2016) for Tyrosine Hydroxylase, for reference.

Below are the validation steps listed by each manufacturer. Furthermore, the expression patterns obtained in this study were compared to previously published images, as referenced by the manufacturer, to confirm accuracy and specificity.

Swant used the following to validate the Calbindin D-28K monoclonal mouse antibody: "McAB 300 reacts specifically with calbindin D-28k on immunoblots of extracts of tissue originating from human, monkey, guinea pig, rabbit, rat, mouse and chicken. McAB 300 does not cross-react with calretinin or other known calcium binding-proteins. McAB 300 specifically stains the 45Ca-binding spot of calbindin D-28k (MW 28'000, IEP 4.8) in a two-dimensional gel. In radioimmunoassay it detects calbindin D-28k with a sensitivity of 10 ng/assay and an affinity of 1.6 x 1012 L/M.McAB 300 against calbindin D-28k specifically localizes calbindin using free-floating or mounted sections of brain, kidney and pancreas of probably all vertebrates with the exception of fishes. In addition, antibody CB300 immunolabels a subpopulation of neurons in the normal brain with high efficiency, but does not stain in the brain of calbindin D-28k knock out mice. References include Celio et al. (1990), Kretsinger et al. (1981), Garcia-Segura et al. (1984), and Airaksinen et al. (1997)."

EMD Millipore used the following to validate the NeuN anti-rabbit antibody: "Anti-NeuN (rabbit polyclonal), Cat. No. ABN78, is rabbit polyclonal antibody that detect NeuN and is tested for use in Immunocytochemistry, Immunohistochemistry (Paraffin), and Western Blotting. Quality assurance was evaluated by Western Blotting in Mouse E16 Brain lysate. 0.5 µg/mL of this antibody was detected NeuN in Mouse E16 Brain lysate. References include Baek et al. (2015), Mellott et al. (2014), Ataka et al., (2013), and Najm et al. (2011)."

Wako used the following to validate the Iba1 anti-rabbit antibody: "Wako has launched rabbit polyclonal antibodies raised against a synthetic peptide corresponding to the Iba1 carboxy-terminal sequence, which was conserved among human, rat and mouse Iba1 protein sequences. These antibodies are specifically reactive to Iba1. Quality assurance was evaluated by Western Blotting (0.5-1 ug/mL). References include Imai et al. (1996), Ito et al. (1998), Ohsawa et al. (2001), and Kanazawa et al. (2002)."

DAKO used the following to validate the GFAP anti-rabbit antibody: "The antibody has been solid-phase absorbed with human and cow serum proteins. In crossed immunoelectrophoresis using 50 µL antibody per cm2 gel area, no reaction with 2 µL human plasma and 2 µL cow serum is observed. The antibody shows one distinct precipitate (GFAP) with cow brain extract. In indirect ELISA, the antibody shows no reaction with human plasma and cow serum. GFAP shows 90-95% homology between species (5), and as demonstrated by immunohistochemistry, the antibody reacts strongly with human GFAP. A reference includes Eng et al. (2000)."

Covance used the following to validate the NFH anti-mouse antibody: "This antibody is effective in immunoblotting (WB) and immunofluorescence (IF). Each lot of this antibody is quality control tested by Western blotting. References include Graeves et al. (2015), Zappulo et al. (2017), Ciolli et al. (2019), and Zhang et al. (2019)."

Millipore Sigma used the following to validate the Tyrosine Hydroxylase anti-rabbit antibody: "Anti-Tyrosine Hydroxylase Antibody detects level of TH and has been published and validated for use in ELISA, IF, IH, IH(P), IP and WB. Quality is routinely evaluated by Western Blot on PC12 lysates. By western blot, AB152 selective labels a single band at approximately 62kDa (reduced) corresponding to Tyrosine Hydroxylase. It is expected that the antibody will react with most mammalian and many non-mammalian species. Brain tissue (corpus striatum, sympathetic nerve terminals, and adrenal glands) were used as the positive control. The liver was used as a negative control. References include Van Kampen et al. (2015), Wen et al. (2014), Bourque et al. (2013), and Ladewig et al. (2012)."

Abcam used the following to validate the anti-slow skeletal myosin heavy chain anti-mouse antibody: "Mouse monoclonal Slow Skeletal Myosin Heavy chain antibody [NOQ7.5.4D]. Validated in WB, ELISA, IHC, RIA, EM, ICC/IF and tested in Mouse, Rat, Sheep, Rabbit, Goat, Chicken, Guinea pig, Hamster and more. This product has been referenced in Chaudhary et al. (2019) and Satoh et al. (2019)."

Abcam used the following to validate the anti-fast skeletal myosin heavy chain anti-rabbit antibody: "This antibody gave a positive signal in the following lysates: Skeletal Muscle (Human) Tissue Lysate; Skeletal Muscle (Mouse) Tissue Lysate; Skeletal Muscle (Rat) Tissue Lysate. Validated by western blotting. References include Wu et al. (2019) and Edman et al. (2019)."

Abcam used the following to validate the laminin anti-rabbit antibody: "Rabbit polyclonal to Laminin. In dot blot immunoassay this antibody does not react with Fibronectin, Vitronectin, Collagen IV, or Chondroitin sulfate types A, B, and C. Postive control, IHC-P: Human skin tissue; mouse skin neoplasia tissue. IHC-Fr: Mouse skin, placenta, anterior tibialis skeletal muscle and testis tissue. References include Zambaiti et al. (2019) and Vaickus et al. (2019)."

Animals and other organisms

Policy information about studies involving animals; ARRIVE guidelines recommended for reporting animal research

Laboratory animals	The Car8wdl mice (Stock #004625) and the C57BLKS/J control background strain were purchased from The Jackson Laboratory (Bar Harbor, ME). The L7Cre;Vgatflox/flox and the Vgatflox/flox control littermates were genetically engineered, as previously described by Lewis et al. (2004) and White et al. (2014). Note that in the literature, L7 is also referred to as Pcp2 (Purkinje cell protein 2) and Vgat is referred to as Slc32a1 (solute carrier family 32 member 1). Mice of both sexes and aged P30, P60-P120, and P150 were studied.
Wild animals	The study did not involve wild animals.
Field-collected samples	The study did not involve samples collected from the field.
Ethics oversight	All animal studies were carried out under an approved IACUC animal protocol according to the institutional guidelines at Baylor College of Medicine (BCM).

Note that full information on the approval of the study protocol must also be provided in the manuscript.
